# The HLA paradox across populations in multiple sclerosis progression: a systematic review

**DOI:** 10.1186/s12883-026-05100-3

**Published:** 2026-06-24

**Authors:** Zhila Maghbooli, Erfan Nasir, Fatemeh Mahdavinasab, Mohammad Ali Sahraian, Arash Shirvani

**Affiliations:** 1 Multiple Sclerosis Research Center, Neuroscience Institute, Tehran University of Medical Sciences, Tehran, Iran; 2 Tehran University of Medical Sciences, Tehran, Iran; 3 Section of Endocrinology, Diabetes, Nutrition and Weight Management, Department of Medicine, Boston University Chobanian & Avedisian School of Medicine, Boston, MA 02118 USA

**Keywords:** Multiple sclerosis, Human leukocyte antigen, HLA-DRB1*15:01, Disease progression, Disability, Magnetic resonance imaging, Systematic review, Meta-analysis

## Abstract

**Background:**

Multiple sclerosis (MS) exhibits heterogeneity in disease courses. Although HLA DRB1*15:01 represents the strongest genetic risk factor for MS susceptibility, conferring approximately threefold increased risk, whether this allele similarly influences disease progression remains contentious.

**Methods:**

This systematic review was conducted to clarify the relationship between HLA polymorphisms and the course of multiple sclerosis from inception through November 2025. A three-level random effects meta-analysis was used to account for statistical non-independence. Study quality was evaluated using Q GENIE criteria.

**Results:**

In total, 56 eligible studies were selected, comprising 22,145 participants; 35 studies contributed 276 effect sizes to quantitative synthesis. The pooled estimate indicated a modest overall association between HLA variants and MS progression (Odds Ratio (OR) 1.30, 95% CI 1.09 to 1.55, *p* = 0.003). HLA DRB1*15 showed no significant association with disease progression (OR 1.10, 95% CI 0.79 to 1.51, *p* = 0.57). Exploratory subgroup analysis revealed significant regional heterogeneity (QM = 10.48, *p* = 0.033): Asian (OR 1.70, *p* < 0.001) and North American (OR 1.95, *p* < 0.001) studies suggested significant effects, whereas European studies showed no effect (OR 1.02, *p* = 0.92). Three alleles demonstrated significant adverse associations: HLA DRB1*04 (OR 1.79, 95% CI 1.33 to 2.42), HLA DRB1*09 (OR 4.21, 95% CI 1.93 to 9.18), and HLA DRB1*03 (OR 1.44, 95% CI 1.01 to 2.05).

**Conclusion:**

This systematic review suggests that multiple sclerosis susceptibility and progression appear governed by distinct genetic architectures.

**Supplementary Information:**

The online version contains supplementary material available at https://doi.org/10.1186/s12883-026-05100-3.

## Background

Multiple sclerosis (MS) is a chronic, immune-mediated demyelinating disease of the central nervous system (CNS) affecting approximately 2.8 million individuals worldwide [1]. Despite decades of research, substantial clinical heterogeneity persists in long-term disability trajectories, with some patients experiencing slowly progressive disease over decades, while others rapidly accumulate neurological disability [2]. Understanding the genetic basis of this phenotypic diversity remains an urgent clinical priority for precision prognostication and therapeutic stratification.

MS pathogenesis arises from a complex interplay between genetic susceptibility and environmental exposures, with an estimated heritability of ~ 48% [3]. The human leukocyte antigen (HLA) region, particularly HLA Class II molecules, constitutes the strongest genetic determinant of MS susceptibility. HLA-DRB1*15:01 confers a strong effect size, increasing MS risk with an average odds ratio of 3.08 in European populations [4].

However, the genetic architecture of MS susceptibility versus disease progression may represent distinct biological phenomena. While susceptibility alleles influence whether individuals develop MS, progression alleles may modulate the burden of CNS inflammation, remyelination capacity, and axonal neuroprotection independently of initial disease onset [5, 6]. Few systematic syntheses have comprehensively examined HLA associations with progression-specific outcomes (disability accrual, MRI lesion burden, and clinical relapse rates) [6–8]. However, these earlier analyses shared a critical methodological limitation: they treated multiple effect sizes derived from the same study populations as independent observations. This approach artificially inflates precision and can produce misleading conclusions.

This systematic review and meta-analysis aim to: (1) synthesize evidence from studies examining HLA associations with MS progression, (2) quantify effects through rigorous meta-analytical approaches, (3) conduct a comprehensive quality assessment to identify methodological limitations, and (4) evaluate geographic heterogeneity in HLA effects.

## Methods

This systematic review and meta-analysis were conducted according to the Preferred Reporting Items for Systematic Reviews and Meta-Analyses (PRISMA) 2020 guidelines [9] and registered prospectively in the International Prospective Register of Systematic Reviews (PROSPERO: CRD420251180977). The comprehensive methodology integrated traditional systematic review techniques with advanced computational approaches, including artificial intelligence-assisted screening and quality assessment tools.

### Literature search strategy

A comprehensive literature search was conducted across three major biomedical databases: PubMed/MEDLINE, Scopus, and Web of Science, covering publications from their inception through November 1, 2025. The search strategy was developed through an iterative refinement process using Medical Subject Headings (MeSH) terms and text words related to multiple sclerosis progression and human leukocyte antigen polymorphisms. It combined controlled vocabulary and free-text terms as follows: (("HLA"[Title/Abstract] OR "human leukocyte antigen"[Title/Abstract] OR "major histocompatibility complex"[Title/Abstract] OR "MHC"[Title/Abstract]) AND ("progress*"[Title/Abstract] OR "disability"[Title/Abstract] OR "severity"[Title/Abstract] OR "prognosis"[Title/Abstract]) AND "multiple sclerosis"[Title/Abstract]). Filters were applied to restrict results to human studies published in English, while excluding reviews, meta-analyses, clinical trials, and conference abstracts to focus on original observational research.

### Study selection criteria

The Population, Intervention, Comparison, and Outcome (PICO) structure guided the construction of study selection criteria. The target population was patients diagnosed with multiple sclerosis, using either the Poser [10], McDonald [11], or revised McDonald criteria [12], with a focus on primary progressive MS (PPMS) and secondary progressive MS (SPMS). Studies were considered eligible if they explored the association between HLA class I or II genotypes and markers of disease progression. Eligible study types included cross-sectional, case–control, and cohort studies that reported quantitative data on HLA allele frequencies and their relationship to disease progression and clinical outcomes. Studies were excluded if they did not differentiate between the progressive and relapsing–remitting courses of the disease, or if they only focused exclusively on disease susceptibility without progression information.

Two authors independently screened titles and abstracts. Full-text articles were retrieved for potentially eligible studies based on predetermined criteria.

### Data extraction process

A standardized approach to data extraction was developed and piloted on five randomly selected studies from the search results. Data extraction was performed independently by two authors using a structured electronic form developed in Microsoft Excel. Extracted variables included study characteristics (first author, publication year, country, study design, sample size), participant demographics (age at onset, gender distribution, disease duration), diagnostic criteria employed (Schumacher, Poser, or McDonald criteria versions), HLA typing method (serological, polymerase chain reaction-Sequence-specific primer (PCR-SSP), sequence-based typing, or next-generation sequencing), and clinical outcome measures including Expanded Disability Status Scale (EDSS) scores, Multiple Sclerosis Severity Score (MSSS), magnetic resonance imaging parameters, and disease course classification. In this systematic review, the term "MS progression" refers primarily to disability accumulation, operationalized as an increase in EDSS or MSSS score, and to disease course (progressive MS [SPMS/PPMS] vs. RRMS).

Where studies reported multiple progression outcomes or examined several alleles, we extracted all relevant effect sizes for multilevel analysis. In cases where original data were unavailable, the effect sizes with confidence intervals were computed from reported frequencies, means, and standard deviations using statistical transformation methods. For studies reporting median values, means were estimated using the method described by Wan and colleagues [13], accounting for sample size and data distribution.

### Quality assessment

The methodological quality of the included studies was evaluated using the Quality of Genetic Studies (Q-GENIE) [14] tool, a validated instrument specifically designed for genetic association studies. The Q-GENIE assessment comprises eleven domains: study rationale and design, ascertainment of genetic variants, technical quality of genotyping, non-technical aspects of laboratory quality, sample size and power calculations, control of bias and confounding, appropriateness of statistical methods, testing for Hardy–Weinberg equilibrium, replication strategy, validity of outcome assessment, and transparency of reporting.

The domains are scored on a seven-point Likert scale. The total scores were categorized as high quality (≥ 60), moderate quality (40–59), or low quality (< 40). Quality assessment was performed independently by two investigators with expertise in genetics, yielding excellent agreement (intraclass correlation coefficient = 0.91). The assessment focused on critical appraisal of: (1) HLA typing methodology (resolution level, error rates); (2) population ancestry adjustment (self-reported vs. genomic markers); (3) multiple testing corrections (Bonferroni, false discovery rate); (4) outcome definition consistency (EDSS assessment protocols, MRI analysis standardization); (5) study design (prospective vs. retrospective, longitudinal follow-up duration).

### Statistical analysis

The primary methodological challenge in conducting this meta-analysis stems from the structure of the available data. Individual studies frequently presented multiple effect sizes from the same population. This means that the data points are correlated, and observations cannot be treated as independent without violating fundamental statistical assumptions.

To address this difficulty, three-level random effects models were used with the “rma.mv” function in the metafor package for R version 4.3.0. This hierarchical framework splits variance into sampling error within individual effect sizes (level 1), heterogeneity among effect sizes from the same study (level 2), and heterogeneity across different studies (level 3).

To test robustness, sensitivity analyses were done using robust variance estimation methods with cluster-robust standard errors. Additionally, study-aggregated analysis was carried out by computing inverse variance-weighted means within each study.

To assess heterogeneity in effect sizes, variance component decomposition, the Cochran Q, I-squared (I^2^), tau-squared tests, and 95% prediction intervals were used. For subgroup analysis, categorical moderators were used in the three-level frameworks. QM statistics were used for moderator analysis.

For publication bias analysis, funnel plots, Egger’s regression, Begg’s rank correlation tests, and trim-and-fill estimation analysis were performed.

## Results

### Literature search and study characteristics

**Fig. 1:**
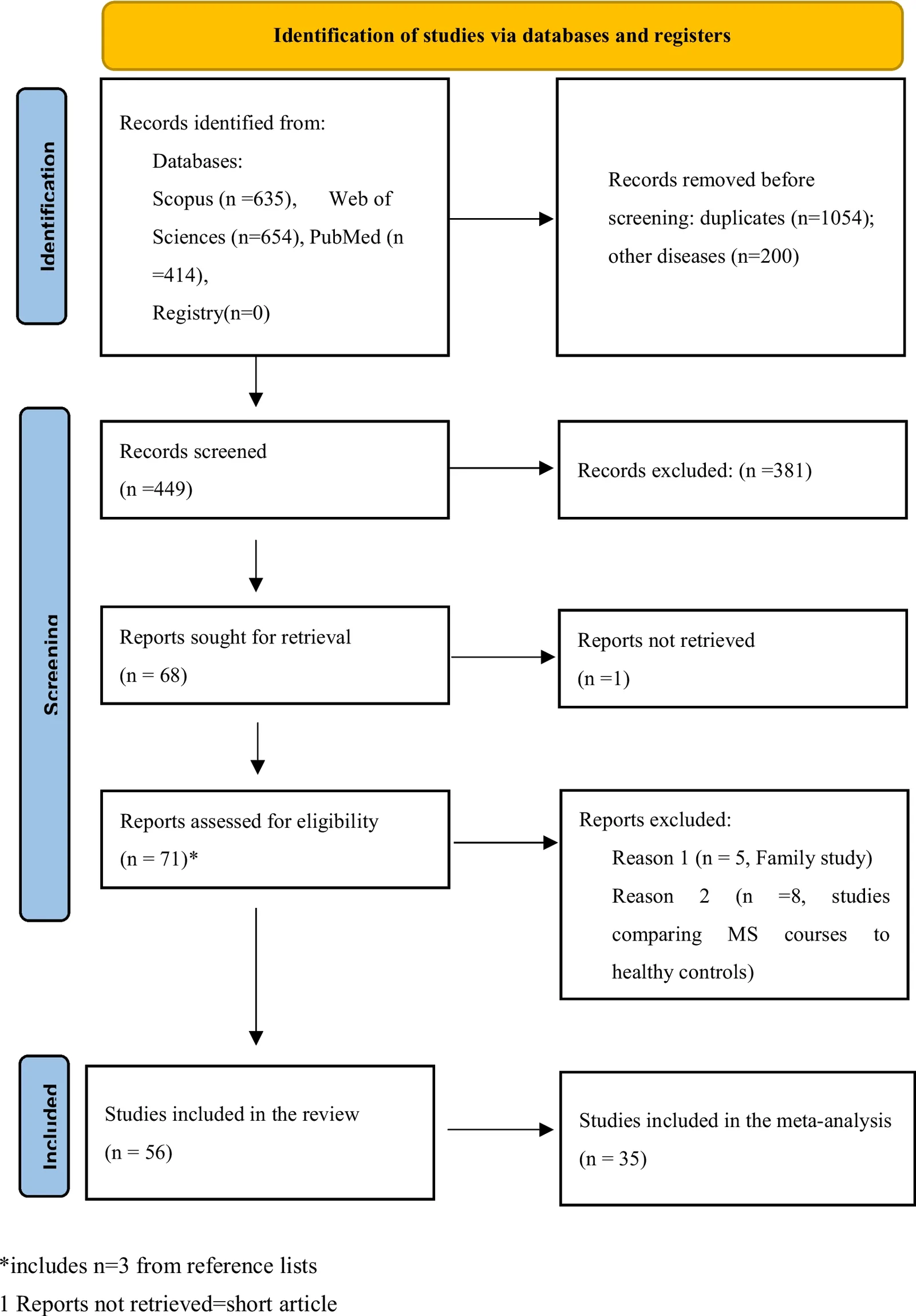
PRISMA 2020 flow diagram of study selection. Flow diagram summarizes identification, screening, eligibility assessment, and inclusion of studies evaluating associations between HLA polymorphisms and MS progression. The figure reports the number of records identified in each database, duplicates removed, records excluded at the title/abstract and full-text stages, and the final 35 studies included in quantitative synthesis

The database searches retrieved 2,847 records. After duplicate removal, 2,313 unique records underwent the screening stage. After evaluating full texts of 171 potentially eligible articles, 56 met the inclusion criteria for qualitative synthesis [15–70], with 35 providing enough quantitative data for inclusion in the meta-analysis [15–20, 24–26, 28–31, 34, 39, 41, 42, 48, 49, 51–58, 60, 61, 63, 66–70]. Figure 1 shows the flow chart describing the study selection process.

The selected studies spanned publication years 1979 through 2025, representing over four decades of research. The geographic distribution included European (n = 32, 57.1%) [16, 20, 23, 24, 32, 34, 39, 43, 45–50, 52, 54, 55, 57–61, 63, 64, 66–72], North American (n = 10, 17.9%) [17, 22, 26, 31, 36, 38, 41, 42, 44, 65], Middle Eastern (n = 7, 12.5%) [21, 28, 33, 35, 53, 56], Asian (n = 3, 5.3%) [18, 25, 30], and other regions (n = 4, 7.1%) [19, 29, 40, 51]. In total, these studies originated from 41 countries. Cumulative enrollment reached 22,145 MS patients (Table 1).

**Table 1:** Characteristics of included studies

Characteristic	N	Details
Total Studies	56	Qualitative synthesis
Studies in Meta-analysis	35	276 effect sizes
Total Participants	22,145	Range: 30–8,060
Geographic Region	N(%)	
European	32 (57.1%)	UK, France, Sweden, Spain, etc
North American	10 (17.9%)	USA, Canada
Middle Eastern	7 (12.5%)	Iran, Saudi Arabia, Morocco
Asian	3 (5.4%)	Japan, Korea, Taiwan
Other (Australian, S. American, Mixed)	4 (7.2%)	
MS Diagnostic Criteria Era		
Pre-McDonald (Poser/Schumacher)	27 (48.2%)	Studies before 2001
McDonald 2001–2010	19 (33.9%)	Original/2005 revision
McDonald 2010+	10 (17.9%)	2010, 2017 revisions
HLA Typing Method		
Molecular (PCR-based)	35 (62.5%)	PCR-SSP, PCR-SSO, PCR–RFLP
Sequencing-based	10 (17.9%)	Sanger, NGS
Serological	7 (12.5%)	Lymphocytotoxicity assays
Not reported	4 (7.1%)	
Study Design		
Case–control	26 (46.4%)	
Cross-sectional	17 (30.4%)	
Cohort	13 (23.2%)	

*Abbreviations*: *MS* multiple sclerosis, *PCR* polymerase chain reaction, *SSP* sequence-specific primers, *SSO* sequence-specific oligonucleotides, *NGS* next-generation sequencing

### Quality assessment

The quality scores ranged from 40 to 77, with 31 studies (88.6%) classified as high quality and 4 (11.4%) as moderate quality. There were no low-quality studies that met the inclusion criteria for quantitative synthesis. Common methodological limitations of the studies included inadequate reporting of Hardy–Weinberg equilibrium testing (42%), insufficient adjustment for population stratification (38%), and failure to apply multiple testing corrections (31%). Studies employing molecular typing methods and McDonald 2010+ criteria generally had higher quality scores. There was a significant improvement in study quality over time (p for trend < 0.001) (Table 2).

**Table 2:** Q-GENIE Quality Assessment Summary for Meta-Analysis Studies (n = 35)

Quality Category	Studies (N)	Effect Sizes	% of Total
High Quality (Q-GENIE ≥ 60)	31	227	88.6%
Moderate Quality (40–59)	4	49	11.4%
Low Quality (< 40)	0	0	0%

### Overall meta-analysis

**Fig. 2:**
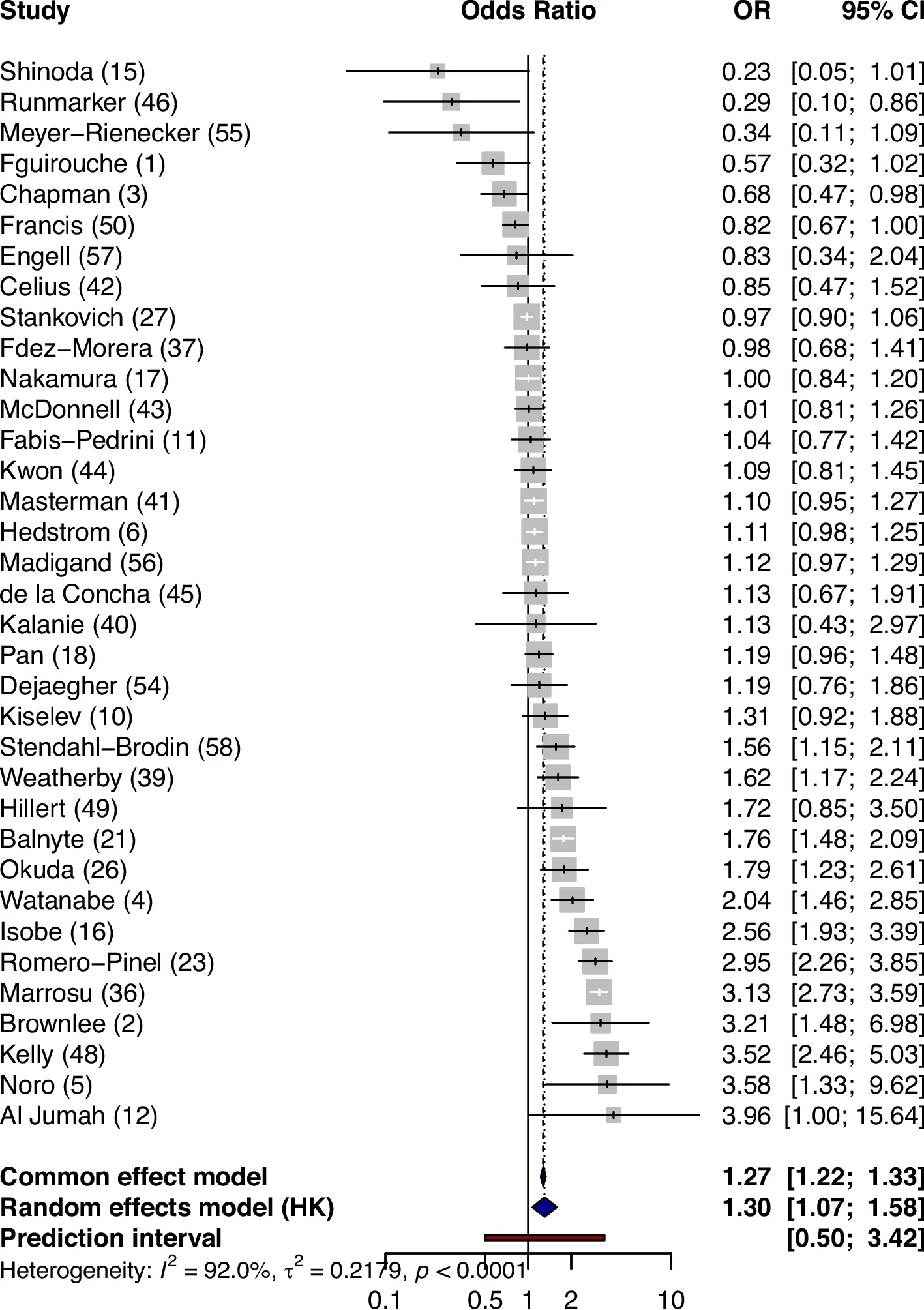
Forest Plot of Overall HLA Association with MS Progression. Forest plot displaying study-aggregated odds ratios and 95% confidence intervals for the association between HLA genetic variants and MS progression (k = 35 studies). Studies are ordered by effect size. The pooled random effects estimate (diamond) shows OR = 1.30 (95% CI: 1.07–1.58). Prediction interval (red line) indicates the range of expected true effects in future studies (0.51–3.30). Substantial heterogeneity is evident (I^2^ = 92%, τ^2^ = 0.218)

The primary three-level model incorporating 276 effect sizes from 35 studies yielded a pooled odds ratio (OR) of 1.30 (95% CI 1.09 to 1.55, *p* = 0.003). The result revealed a modest but statistically significant association between HLA variants and MS progression. Robust variance estimation confirmed this finding (OR 1.30, 95% CI 1.08 to 1.56, *p* = 0.006). The analysis of the aggregated studies showed the same result as the three-level analysis (OR 1.30, 95% CI 1.07 to 1.58, *p* = 0.010) (Table 3, Fig. 2).

**Table 3:** Overall Meta-Analysis Results: Comparison of Analytical Methods

Analysis Method	OR	95% CI	*P*-value	k (studies)
Three-level model (primary)	1.30	1.09–1.55	0.003	35
Robust variance estimation	1.30	1.08–1.56	0.006	35
Study-aggregated	1.30	1.07–1.58	0.010	35

*OR* Odds ratio, *CI* Confidence interval, *k* Number of independent studies. The three-level model accounts for effect sizes (Level 1) nested within studies (Level 2) with between-study variance (Level 3). All methods yield consistent estimates, supporting robustness of findings

### Heterogeneity assessment

Variance decomposition showed that the total percentage of heterogeneity was made up of 65.2% due to within-study variation (reflecting variation among different alleles and outcomes examined in the same populations), while the remaining 34.8% represented between-study heterogeneity. The aggregated analysis showed an I^2^ of 92.0% and a 95% prediction interval between OR 0.51 and 3.30.

### HLA allele specific analyses

The most striking result was found in allele-specific analyses. HLA DRB1*15, despite being the canonical MS susceptibility allele and the most frequently studied variant in our dataset (34 effect sizes from 22 studies), showed no significant association with disease progression (OR 1.10, 95% CI 0.79 to 1.51, *p* = 0.57). The confidence interval showed no clinically meaningful effects in either direction.

**Fig. 3:**
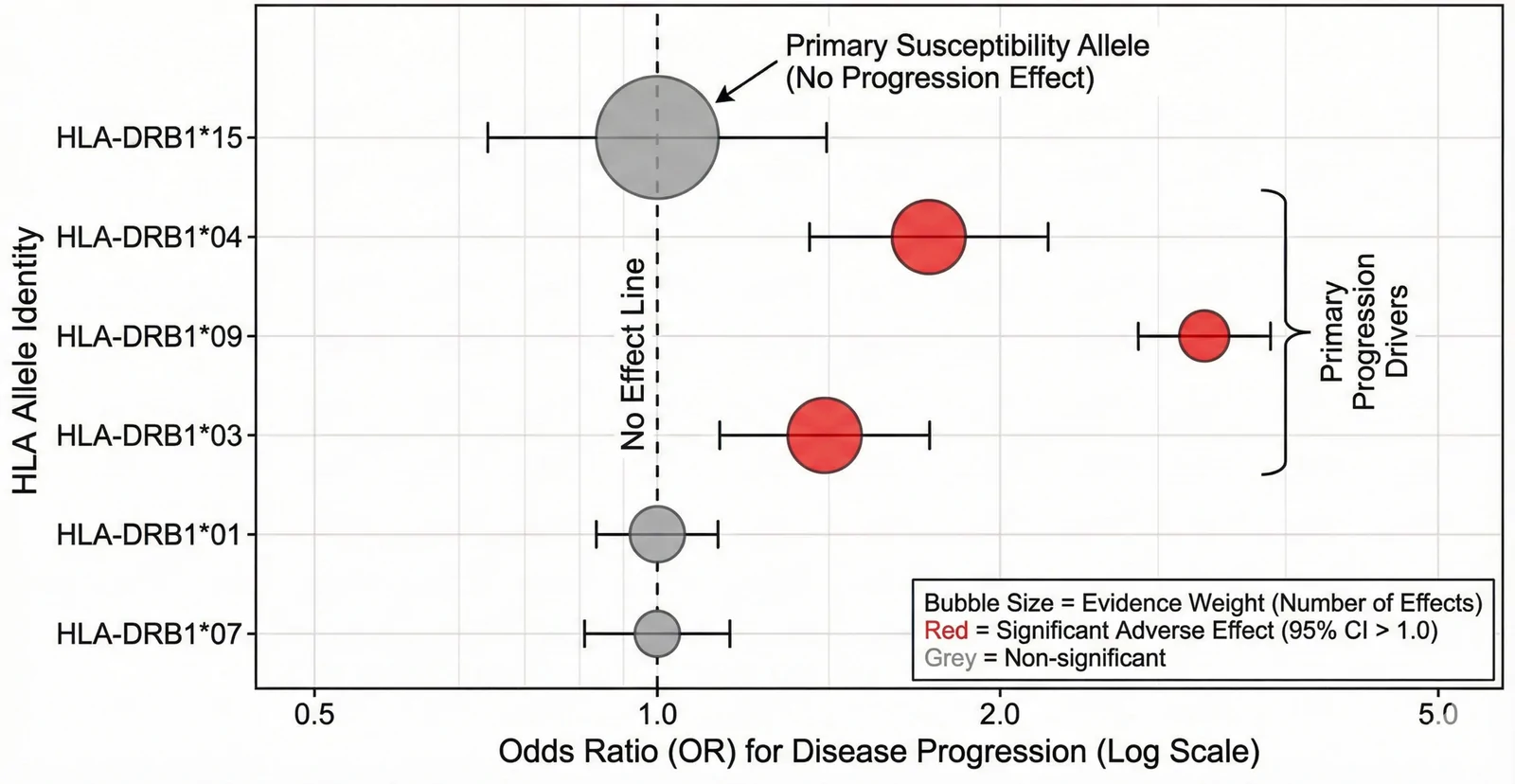
The Dissociation of Multiple Sclerosis Susceptibility and Progression Genetics. This bubble plot illustrates the fundamental dissociation between genetic susceptibility and disease course. The X-axis represents the pooled Odds Ratio (OR) for MS disease progression (log scale), and bubble size is proportional to the number of effect sizes contributing to the estimate. HLA-DRB1*15, the canonical susceptibility allele (grey), clusters near the line of no effect (OR = 1.10, *p* = 0.57), indicating it does not influence disability accumulation despite its role in disease initiation. In contrast, HLA-DRB1*09, HLA-DRB1*04, and HLA-DRB1*03 (red) demonstrate significant rightward shifts, identifying them as distinct genetic determinants of aggressive disease course. Error bars represent 95% confidence intervals derived from the three-level random-effects model

**Fig. 4:**
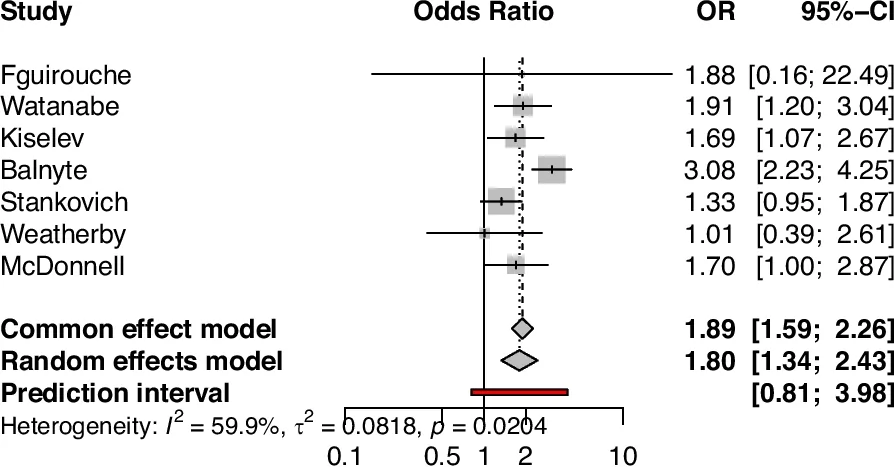
Forest Plot for HLA-DRB1*04 Association with MS Progression. Forest plot displaying study-aggregated odds ratios and 95% confidence intervals for the association between HLA-DRB1*04 and MS progression (k = 7 studies, 8 effect sizes). The pooled estimate shows a significant adverse association (OR = 1.79, 95% CI: 1.33–2.42, *p* < 0.001) with moderate heterogeneity (I^2^ = 59.9%)

**Fig. 5:**
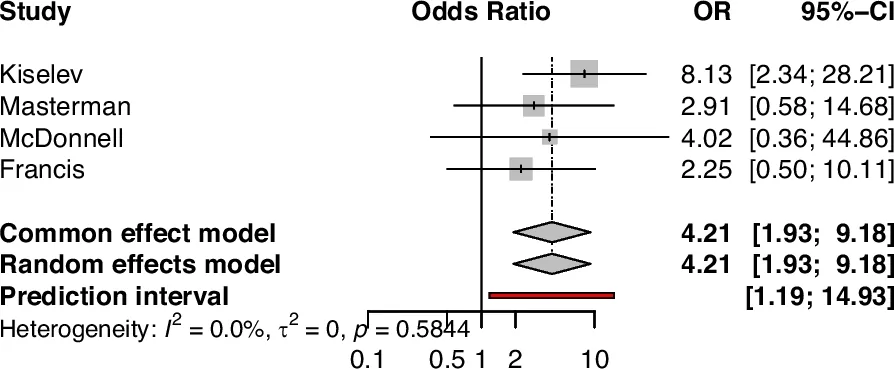
Forest Plot for HLA-DRB1*09 Association with MS Progression. Forest plot displaying odds ratios and 95% confidence intervals for the association between HLA-DRB1*09 and MS progression (k = 4 studies, 5 effect sizes). The pooled estimate shows the strongest effect among all alleles (OR = 4.21, 95% CI: 1.93–9.18, *p* < 0.001) with no heterogeneity (I^2^ = 0%)

**Fig. 6:**
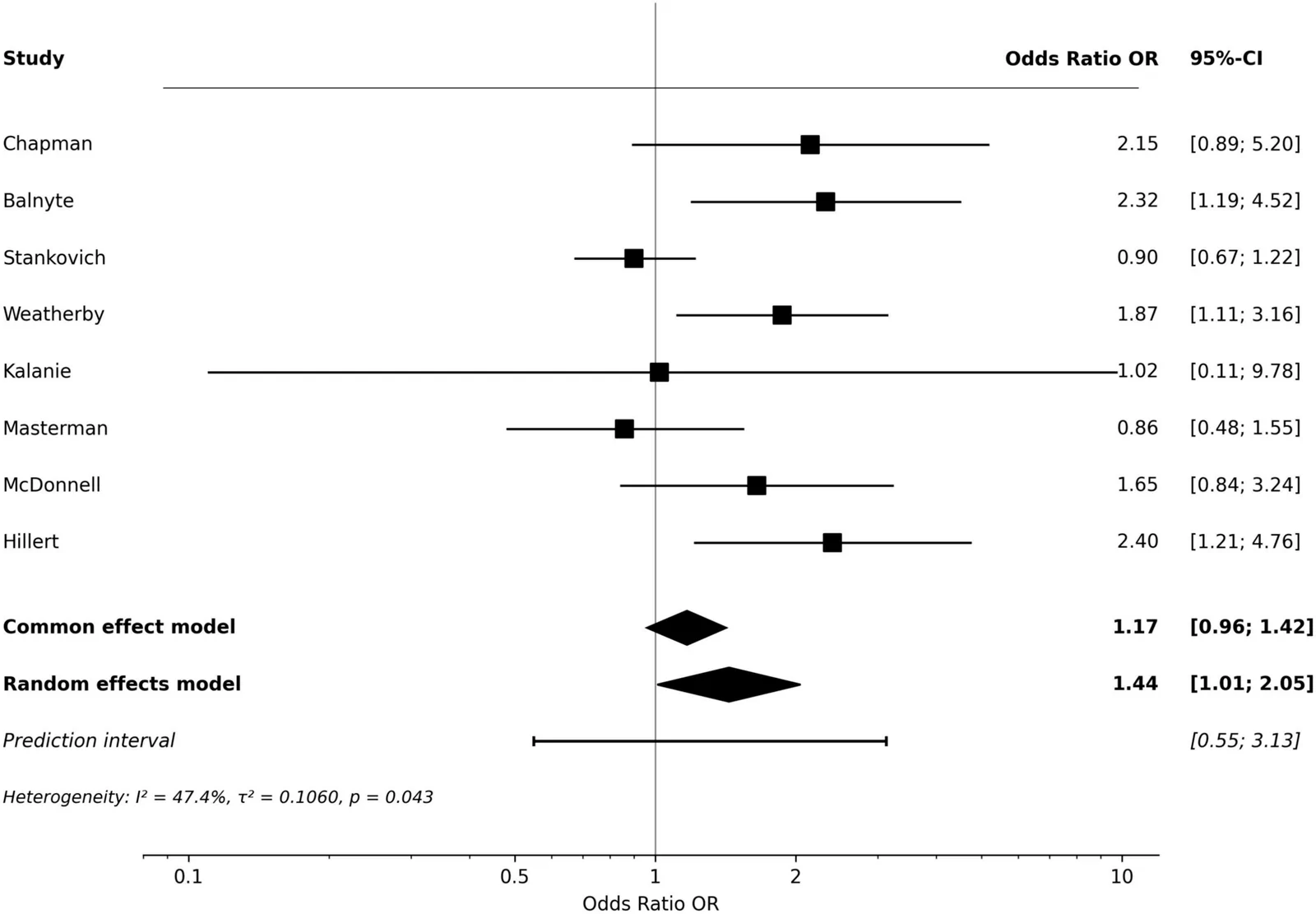
Forest Plot for HLA-DRB1*03 Association with MS Progression. Forest plot displaying odds ratios and 95% confidence intervals for the association between HLA-DRB1*03 and MS progression (k = 8 studies, 12 effect sizes). The pooled estimate shows a modestly significant association (OR = 1.44, 95% CI: 1.01–2.05, *p* = 0.043) with moderate heterogeneity (I^2^ = 47.4%)

Three alleles showed significant adverse associations with progression. HLA DRB1*04 allele showed a robust effect (OR 1.79, 95% CI 1.33 to 2.42, *p* < 0.001) based on 8 effect sizes from 7 studies with moderate heterogeneity (I^2^ = 59.9%). HLA DRB1*09 exhibited the largest effect size (OR 4.21, 95% CI 1.93 to 9.18, *p* < 0.001) with low heterogeneity (I^2^ = 0%), although based on fewer observations. HLA DRB1*03 demonstrated a more modest association (OR 1.44, 95% CI 1.01 to 2.05, *p* = 0.043) (Figs. 3, 4, 5 and 6).

Put all together, these three progression-associated alleles produced substantially larger effects (combined mean OR 2.56) than non-significant alleles, including DRB1*15, DRB1*01, DRB1*07, DRB1*11, and DRB1*13 (combined mean OR 1.50), representing a 71% relative difference (Table 4).

**Table 4:** HLA Allele-Specific Meta-Analysis Results

HLA Allele	Effects	Studies	OR	95% CI	*P*-value	I^2^
DRB1*15	34	22	1.10	0.79–1.51	0.575	—
DRB1*04^*^	8	7	1.79	1.33–2.42	< 0.001	59.9%
DRB1*03^*^	12	8	1.44	1.01–2.05	0.043	47.4%
DRB1*09^*^	5	4	4.21	1.93–9.18	< 0.001	0%
DRB1*01	11	8	0.97	0.57–1.64	0.908	—
DRB1*07	9	7	1.06	0.74–1.52	0.744	—
DRB1*11	7	6	1.00	0.73–1.38	0.988	—
DRB1*13	10	7	0.98	0.73–1.32	0.897	—
HLA-DQ	16	6	1.63	0.75–3.52	0.218	—
HLA-A	7	3	1.13	0.96–1.35	0.145	—

*OR* Odds ratio, *CI* Confidence interval, *I*^2^ Heterogeneity statistic. Three-level models are used for all alleles to account for the clustering of effect sizes within studies. *Effects* Number of effect size estimates, *Studies *Number of unique studies contributing data

^*^Statistically significant (*p* < 0.05)

### Regional heterogeneity in DRB1*15 effects

Exploratory subgroup analysis revealed a significant regional heterogeneity in DRB1*15 progression effects (QM = 10.48, df = 4, p = 0.033). Asian studies (2 studies, 5 effect sizes) suggested a significant adverse association (OR 1.70, 95% CI 1.32 to 2.19, *p* < 0.001). North American studies (3 studies, 6 effect sizes) similarly showed significant effects (OR 1.95, 95% CI 1.36 to 2.78, *p* < 0.001). In contrast, European studies comprising the largest and most robust evidence base (14 studies, 18 effect sizes) demonstrated null effects (OR 1.02, 95% CI 0.64 to 1.63, *p* = 0.92). Middle Eastern studies (3 studies, 5 effect sizes) showed a non-significant trend toward protection (OR 0.58, 95% CI 0.21 to 1.63, *p* = 0.30).

These findings should be interpreted with caution, as the results of Asian and North American subgroups are based on relatively few studies (only 3 to 5), which limits statistical reliability. The European subgroup, despite showing high within-region variance (individual study ORs ranging from 0.12 to 7.88), provides the most stable estimate due to its larger sample size. The overall null association in the primary analysis is observed in European studies (Table 5).

**Table 5:** Regional Subgroup Analysis for HLA DRB1*15 and MS Progression

Region	Studies	Effects	OR	95% CI	*p* value
European	14	18	1.02	0.64 to 1.63	0.92
Asian	2	5	1.70	1.32 to 2.19	< 0.001^*^
North American	3	6	1.95	1.36 to 2.78	< 0.001^*^
Middle Eastern	3	5	0.58	0.21 to 1.63	0.30

Regional heterogeneity test: QM = 10.48, df = 4, *p* = 0.033

^*^Statistically significant. Note that Asian and North American subgroups comprise only 2 to 3 studies each; these findings require cautious interpretation

### HLA class comparison

The association of HLA Class II alleles with disease progression was found to be more significant (mean OR 1.93, 150 effect sizes from 28 studies) than that of HLA Class I alleles (mean OR 1.22, 12 effect sizes from 4 studies), with a 58% relative difference. This pattern suggests that CD4-positive T cell-mediated mechanisms may predominate in driving disability accumulation (Table 6).

**Table 6:** HLA Class I versus Class II Comparison

HLA Class	Effect Sizes	Studies	Mean OR	Relative Difference
Class I	12	4	1.22	Reference
Class II	150	28	1.93	+ 58% versus Class I

Class II alleles present to CD4 + helper T cells; Class I alleles present to CD8 + cytotoxic T cells. The stronger Class II effect suggests CD4 + T cell-mediated mechanisms may predominate in driving disability accumulation

### Temporal evolution

Studies published before 2000 reported larger effect estimates (mean OR 2.32, 111 effect sizes) compared with those published from 2010 onward (mean OR 1.74, 91 effect sizes), representing a 25% attenuation. This trend likely reflects methodological improvements, including the adoption of molecular typing and more rigorous diagnostic criteria. Meta-regression found no statistically significant temporal trend (*p* = 0.66), suggesting the underlying association remained stable (Table 7).

**Table 7:** Temporal Evolution of Effect Estimates

Publication Era	Effect Sizes	Mean OR	Change
Before 2000	111	2.32	Reference
2000 to 2009	74	1.91	18% attenuation
2010 and later	91	1.74	25% attenuation

Effect estimates attenuated over time, likely reflecting methodological improvements, including molecular typing and standardized diagnostic criteria. Meta-regression showed no statistically significant temporal trend (*p* = 0.66)

### Publication bias and sensitivity analyses

**Fig. 7:**
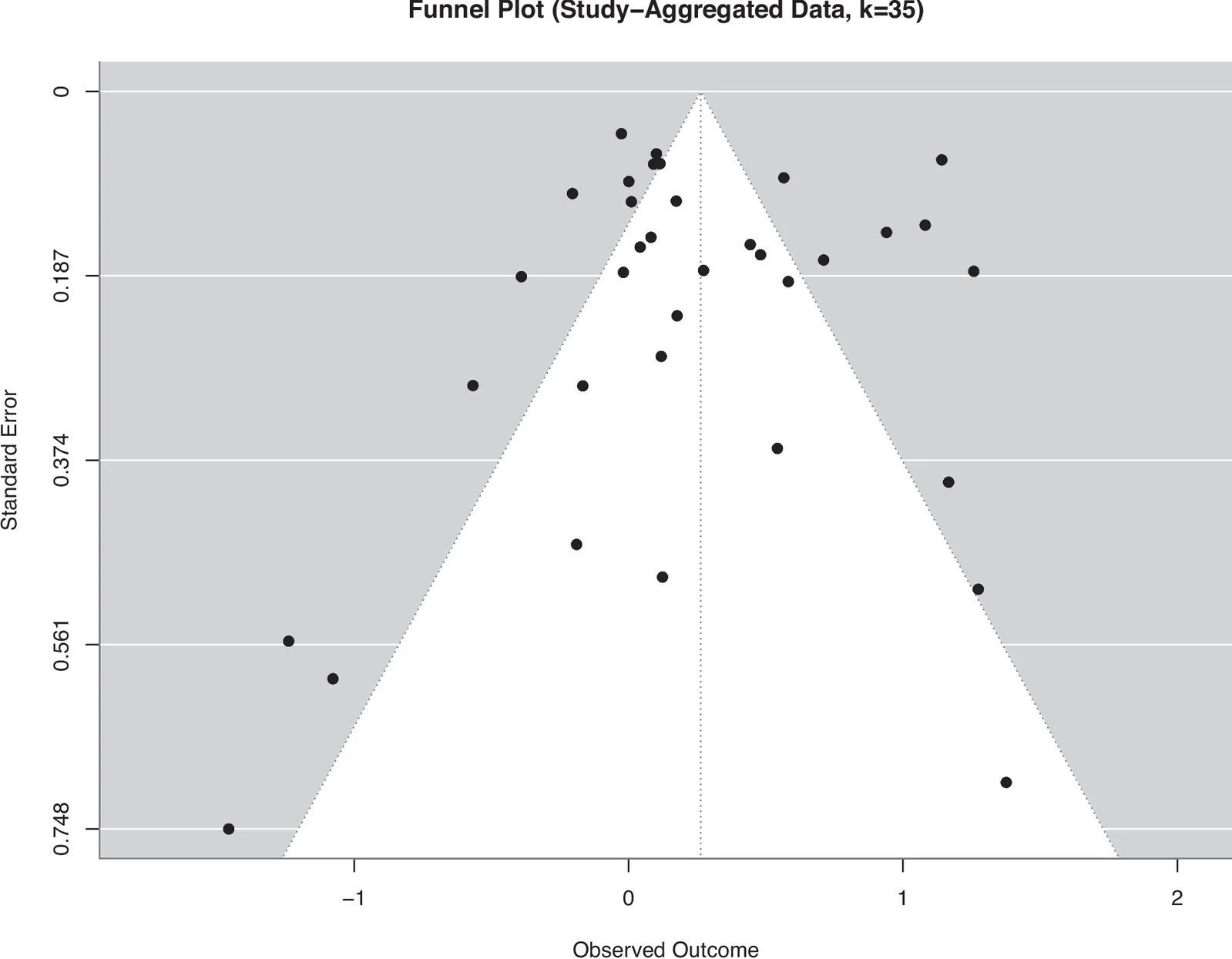
Funnel Plot for Publication Bias Assessment. Funnel plot of log odds ratios versus standard error for studies reporting associations between HLA variants and MS progression (k = 35 studies). Visual inspection shows no substantial asymmetry. Formal tests confirmed absence of publication bias: Egger’s test (*p* = 0.66), Begg’s test (*p* = 0.76). Trim-and-fill analysis estimated 3 potentially missing studies; adjusted estimate remained significant (OR = 1.39, *p* = 0.0006)

Funnel plot inspection showed no asymmetry (Fig. 7). The absence of publication bias was confirmed by Egger regression (t = 0.44, *p* = 0.66) and Begg rank correlation (tau = 0.039, *p* = 0.76). Trim-and- fill analysis estimated three potentially missing studies; the adjusted estimate remained significant (OR 1.39, 95% CI 1.14 to 1.67, *p* = 0.0006).

The leave-one-out sensitivity analysis showed stability. Excluding any single study shifted the pooled estimates minimally, from OR 1.26 to OR 1.33. When Runmarker et al.’s study was excluded, a heavily weighted historical cohort, the estimate was altered by only 0.03. This shows that findings do not depend on any individual study.

## Discussion

This systematic review and multilevel meta-analysis represent the most extensive synthesis to date of HLA effects on MS outcome.

Our findings challenge prevailing assumptions and give novel insights that would have been disregarded in previous studies.

### The susceptibility progression dissociation

The key finding is a clear genetic dissociation of MS susceptibility from that of progression. HLA DRB1*15 has been confirmed to increase MS susceptibility approximately threefold but has not been associated with disease progression yet (OR 1.10, *p* = 0.57). The difference between susceptibility and progression effect sizes for the same allele is too large to attribute to statistical noise.

The finding rests on the most extensive evidence based on our analysis and proved robust across sensitivity analyses.

Weatherby and colleagues reached a similar conclusion over two decades ago, stating that the contribution of HLA in MS is primarily towards onset and initial triggering mechanisms rather than influencing disease progression, chronicity, and severity [51]. Our meta-analysis now confirms this observation with substantially greater statistical power and appropriate multilevel methodology.

### Regional heterogeneity: real or artifact?

The overall analysis demonstrates substantial heterogeneity (I^2^ = 92%). The significant regional heterogeneity we observed for DRB1*15 (*p* = 0.033) merits careful consideration. Asian and North American studies suggested significant progression effects, whereas European studies showed null associations. The non-European subgroups comprise only 2 to 3 studies each, providing limited statistical reliability. The European subgroup, with 14 studies, shows high internal variance with individual study ORs ranging from 0.12 to 7.88.

This variance suggests that factors other than geography, such as outcome definitions, follow-up duration, and patient selection, likely contribute to observed heterogeneity. Previous suggestions of population-specific effects may have reflected these methodological differences rather than true biological heterogeneity.

Possible candidates include differential Epstein-Barr virus exposure, vitamin D status, and linkage disequilibrium with other HLA variants that differ across populations. To address regional heterogeneity, researchers should conduct meta-regression analyses with regional covariates (ethnicity, vitamin D status, and EBV exposure) and perform stratified meta-analyses using granular categories (Northern vs. Southern Europe, East vs. South Asia) rather than broad groups. Additionally, standardize MS progression outcome definitions (EDSS, MSSS, MRI) across studies, recruit larger non-European cohorts for better statistical power, and conduct haplotype or linkage disequilibrium analyses to determine if DRB1*15 effects are confounded by population-specific HLA variants. Until larger non-European cohorts are available and these approaches are implemented, the overall null association derived primarily from European data represents the most reliable estimate, and regional subgroup estimates—particularly from small non-European cohorts—should be interpreted with caution.

### Biological basis for dissociation

The dissociation between DRB1*15 and MS progression can be understood through compartmentalized inflammation. In progressive MS, the inflammatory process gets trapped behind a repaired blood–brain barrier. It works independently of peripheral immune mechanisms [73, 74]. DRB1*15 allele facilitates disease initiation through at least two known pathways: presentation of myelin peptides to autoreactive CD4-positive T cells in the periphery and serving as a co-receptor for Epstein-Barr virus entry into B cells [75–77]. However, once CNS inflammation becomes compartmentalized behind an intact barrier, these peripheral mechanisms are no longer relevant to ongoing neurodegeneration.

Neuropathological studies support this interpretation. Machado Santos and colleagues demonstrated that CD8-positive tissue resident memory T cells predominate in chronic MS lesions, representing a cell population distinct from the peripheral T cells [73], which is influenced by DRB1*15 [77]. Magliozzi and colleagues showed that meningeal B cell follicles, which relate to cortical damage and rapid progression, work independently of peripheral immune activity [78]. Anti-inflammatory therapies targeting peripheral cells show limited efficacy in progressive disease. It is consistent with the concept that compartmentalized inflammation leads to disability accumulation through mechanisms not captured by susceptibility genetics.

### Progression associated alleles: different mechanisms

In contrast to DRB1*15, the alleles we identified as linked to disease progression may influence CNS resident immune responses. HLA DRB1*04 contains the shared epitope structure implicated in tissue-destructive autoimmunity in rheumatoid arthritis [79]. The valine residue at position 86 in the peptide-binding groove changes how antigens are presented. This alteration may continue to lead to chronic inflammation within the CNS compartment. Romero Pinel and colleagues independently reported that DRB1*04 predicts a worse prognosis in Spanish MS patients [37], supporting our meta-analytic finding.

Japanese MS studies provide additional insight. Kikuchi and colleagues found that DRB1*15 carriers have higher CSF immunoglobulin abnormalities reflecting intrathecal B cell activation. In contrast, DRB1*04 carriers show milder CSF changes with distinct progression patterns [30, 80]. These phenotypic differences support the hypothesis that DRB1*15 and DRB1*04 influence MS through basically different biological pathways.

### HLA class effects

The 58% stronger progression effects we observed for Class II compared with Class I alleles have important mechanistic implications. Class II molecules present extracellular antigens to CD4-positive helper T cells and manage inflammatory responses through cytokine secretion. Class I molecules present intracellular peptides to CD8-positive cytotoxic T cells. Our findings suggest that CD4-positive T cell-mediated licensing may be important for progression, even though CD8-positive cells numerically dominate in chronic lesions. It seems that this paradox may reflect the role of CD4-positive cells in providing help to boost CD8-positive cytotoxicity or stimulate microglial activation through unrelated mechanisms.

### A two-stage model

We propose a two-stage model of HLA effects in MS that brings together our findings with the biological literature. In the first stage governing disease initiation, DRB1*15 promotes the activation of autoreactive T cells in the periphery and potentially facilitates EBV-mediated autoimmunity. These cells can cross the blood–brain barrier and cause focal CNS inflammation, manifesting as clinical relapses. In the second stage of disease progression, inflammation compartmentalizes behind a repaired barrier. At this point, alleles like DRB1*04, DRB1*09, and DRB1*03 may preferentially present CNS resident antigens to tissue resident T cells, causing chronic microglial activation and axonal degeneration, independent of peripheral immunity.

This model explains the central paradox: DRB1*15 carriers develop MS more often, but once the disease is established, their progression is no worse than that of non-carriers. This suggests that genetic variants contributing to disease initiation differ from those contributing to disease progression.

### Clinical implications

These findings have direct implications for clinical practice.

First, HLA DRB1*15 status should not inform prognosis. While DRB1*15 is the major genetic risk allele for MS susceptibility, carrying it does not predict a worse disease course. Patients and clinicians should understand that prognostic models incorporating DRB1*15 as a severity marker are likely to be uninformative or misleading.

Second, screening for DRB1*04 and DRB1*09 should be evaluated as potential prognostic markers, particularly when considered in combination. Although DRB1*09 showed a strong association (OR = 4.21), this finding is based on only four studies and five effect sizes, so it should be considered exploratory until replicated. Nevertheless, the higher combined effect of progression-associated alleles compared with non-significant alleles suggests meaningful stratification may be achievable.

Third, the failure of peripheral anti-inflammatory therapies in progressive MS aligns with our genetic findings that DRB1*15 does not predict disease progression. Treatments targeting DRB1*15-mediated mechanisms may be effective for reducing relapses but ineffective for slowing disability accumulation. CNS-penetrant therapies addressing compartmentalized inflammation may be required for progression.

### Limitations

Several limitations warrant acknowledgment.

First, substantial heterogeneity was observed (I^2^ = 92%), likely reflecting biological variation across alleles and outcomes. The wide prediction interval (OR 0.51 to 3.30) suggests a high degree of variability in the HLA alleles based on the progression phenotypes examined. Progression outcomes were heterogeneous, including EDSS milestones, MS severity scores, and MRI endpoints.

Second, most studies inadequately adjusted for population stratification, potentially leading to residual confounding. Evidence for some alleles, particularly DRB1*09, derives from only a few studies and should be considered exploratory. The regional heterogeneity findings, while statistically significant, rely on small non-European samples and require replication in larger cohorts.

Third, protective HLA alleles could influence disease progression, and although this was not the aim of our study, such alleles may act as confounders.

Finally, environmental interactions with HLA genes- such as smoking [81], passive smoking [82], and alcohol consumption [83]- could act as unmeasured confounders in most of the included studies, since these exposures are known to interact with HLA genotype in MS.

### Future directions

Based on our findings, we recommend that future studies begin with targeted high-resolution typing of the progression-associated alleles.

Our meta-analysis provides robust evidence for these specific HLA polymorphisms, making them priority candidates -DRB1*04, DRB1*09, and DRB1*03- for validation and mechanistic studies. Targeted approaches offer several advantages: they are more cost-effective, enable amino acid-level resolution, and allow focused validation in larger cohorts.

Additionally, the finding by DeLuca et al. (2007) that HLA-DRB1*01 is protective against disease progression should be followed up in future studies. The extremes-of-outcome method they used allowed them to identify this allele, whereas other studies may have missed it due to different analytical approaches.

However, genome-wide approaches (such as next-generation sequencing or whole-exome sequencing) should be employed in parallel or in subsequent studies to identify additional genetic determinants beyond the HLA region.

The HLA region is complex, and linkage disequilibrium patterns may obscure other causal variants. Genome-wide approaches will also enable the development of progression-specific polygenic risk scores incorporating both HLA and non-HLA variants.

Future research should prioritize larger, multi-ethnic cohorts with standardized outcome definitions to clarify whether population-specific environmental or genetic factors (e.g., EBV exposure, vitamin D status, HLA linkage disequilibrium) modulate the DRB1*15 effect on MS progression.

## Conclusions

The present systematic review, which comprises a total of 56 studies with over 22,000 MS patients, reveals a fundamental dissociation between susceptibility and progression genetics. The canonical susceptibility allele HLA DRB1*15 does not have predictive value for the clinical course of the disease. The overall null finding was mainly driven by robust evidence from European data. Although the regional heterogeneity was found to be statistically significant, the evidence was based on a limited non-European population and requires cautious interpretation. Instead of DRB1*15, HLA DRB1*04, DRB1*09, and DRB1*03 emerge as the most relevant genetic determinants of disease progression. In addition, Class II alleles showed 58% stronger effects than Class I variants.

The biological basis for this dissociation involves compartmentalized inflammation. DRB1*15 influences peripheral immune activation and disease onset but does not affect the trapped CNS inflammation that causes progressive disability. The genes that increase MS risk are not the same as those that influence its severity.

Future research and clinical translation efforts should focus on progression-specific genetic markers. Understanding the biological distinction between disease initiation and disease course may ultimately reveal new therapeutic targets for preventing disability accumulation in MS.

## Supplementary Information

Supplementary Material 1.

Supplementary Material 2.

## Data Availability

All data generated or analyzed during this study are included in this published article and its supplementary information files.
